# A hybrid omental-parietal peritoneal fibrous sheath: a case report of an unreported cause of recurrent ventriculoperitoneal shunt failure

**DOI:** 10.3389/fmed.2026.1890582

**Published:** 2026-08-14

**Authors:** Jinshen Xie, Jia Wu, Haokun Jiao, Haijun Xia, Yuping Peng, Tiejian Liu

**Affiliations:** Department of Neurosurgery, The Third Affiliated Hospital of Southern Medical University, Guangzhou, Guangdong, China

**Keywords:** distal catheter obstruction, hydrocephalus, peritoneal fibrosis, shunt malfunction, ventriculoperitoneal shunt

## Abstract

**Background:**

Hydrocephalus often requires surgical cerebrospinal fluid diversion, and ventriculoperitoneal (VP) shunting remains one of the most widely used treatments. Distal catheter obstruction is a common cause of ventriculoperitoneal (VP) shunt malfunction. Typical abdominal mechanisms include omental wrapping, adhesions, catheter migration, perforation, and cerebrospinal fluid (CSF) pseudocyst formation. Obstruction caused by a parietal peritoneum–anchored fibrous sheath has rarely been described.

**Case description:**

A 30-years-old man underwent VP shunt placement for communicating hydrocephalus after surgery for intracerebral hemorrhage. He subsequently developed recurrent shunt dysfunction and underwent two distal catheter revisions at an outside hospital within 1 month, with only transient improvement. On admission to our institution, he presented with a tense decompressive craniectomy defect and progressive ventriculomegaly. Abdominal computed tomography showed that the distal catheter coursed toward the right subphrenic region without obvious fluid accumulation. Diagnostic laparoscopy revealed dense omental adhesions and, more importantly, a complete fibrous sheath encasing the distal catheter and fixed to the parietal peritoneum. The sheath formed a blind-ended cavity that isolated the catheter tip and side holes from the absorptive peritoneal surface. No evidence of active shunt infection was identified. Laparoscopic sheath release, distal catheter revision, and repositioning of the catheter away from the abdominal wall restored CSF drainage. Postoperative imaging showed reduced ventriculomegaly and resolution of external brain herniation.

**Conclusion:**

This case illustrates a rare mechanism of distal VP shunt failure caused by hybrid omental–parietal peritoneal fibrous sheath formation. Laparoscopy is valuable for diagnosis, functional confirmation, sheath release, and strategic catheter repositioning.

## 1 Introduction

Ventriculoperitoneal (VP) shunting remains the cornerstone treatment for hydrocephalus. However, mechanical complications continue to affect a considerable proportion of patients and frequently necessitate repeated revision procedures. A recent systematic review and meta-analysis of 38,095 adult shunt insertion procedures reported an overall pooled shunt failure rate of 17%. In the same analysis, obstruction was the most common cause of shunt failure, accounting for 23.2% of all failures, while the distal catheter was the most frequent site of malfunction, accounting for 33.4% (1). These findings further indicate that distal catheter dysfunction represents one of the leading causes of shunt failure, with obstruction being its most common mechanism (2–4). Established abdominal causes of distal VP shunt obstruction include omental wrapping, intra-abdominal adhesions secondary to previous surgery or peritonitis, catheter migration, and cerebrospinal fluid (CSF) pseudocyst formation (5–7). In these conventional scenarios, the obstructing tissues are typically mobile within the peritoneal cavity and demonstrate relatively well-characterized pathological processes. In contrast, obstruction caused by fixed blind-ending fibrous chambers anchored to the abdominal wall has rarely been described. Here, we present a rare case of recurrent VP shunt malfunction caused by a hybrid fibrous structure involving both adherent omentum and a dense fibrotic sheath apparently originating from the parietal peritoneum within the right subphrenic space. The lesion formed a sealed blind cavity that isolated the distal catheter tip from the absorptive peritoneal surface, resulting in repeated distal shunt failure.

## 2 Case description

**FIGURE 1:**
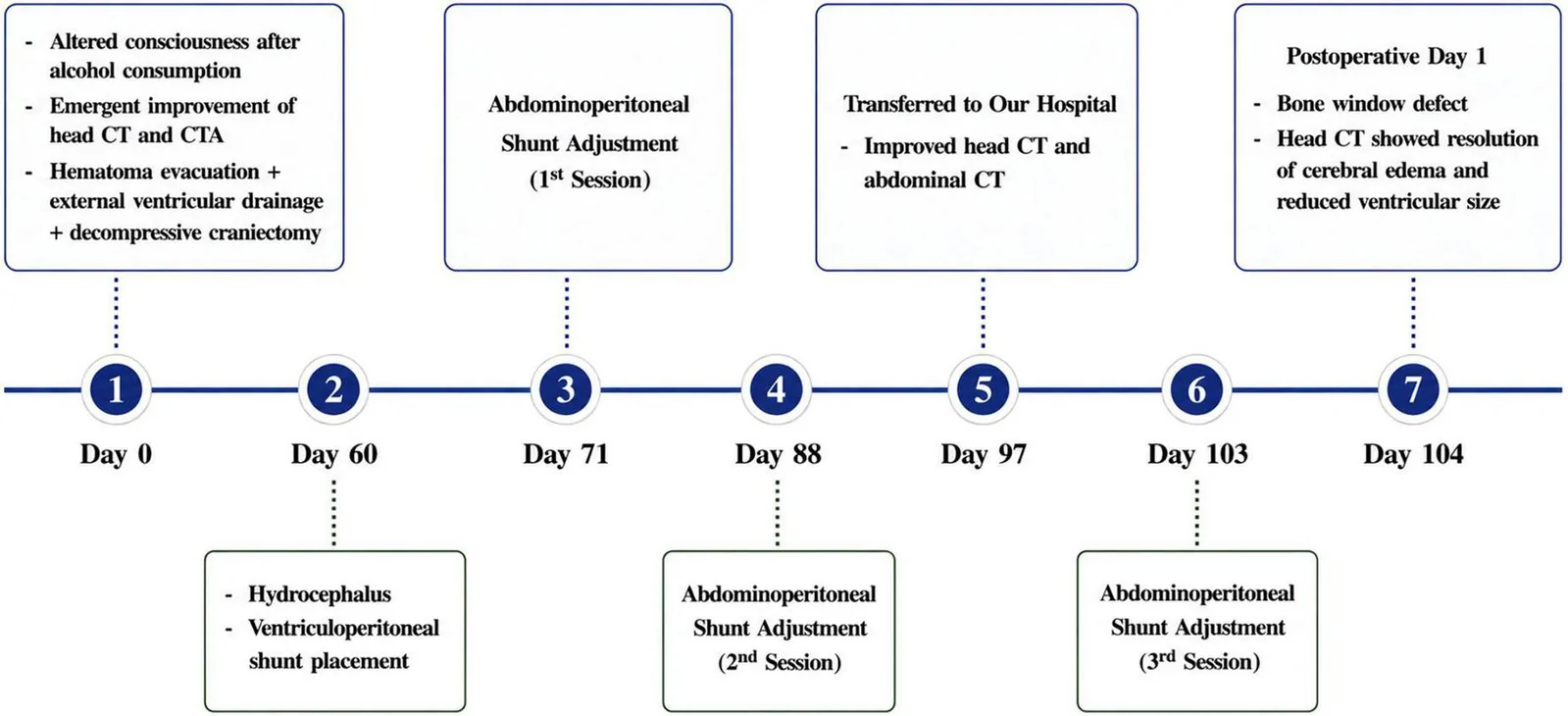
Treatment timeline of the patient.

**FIGURE 2:**
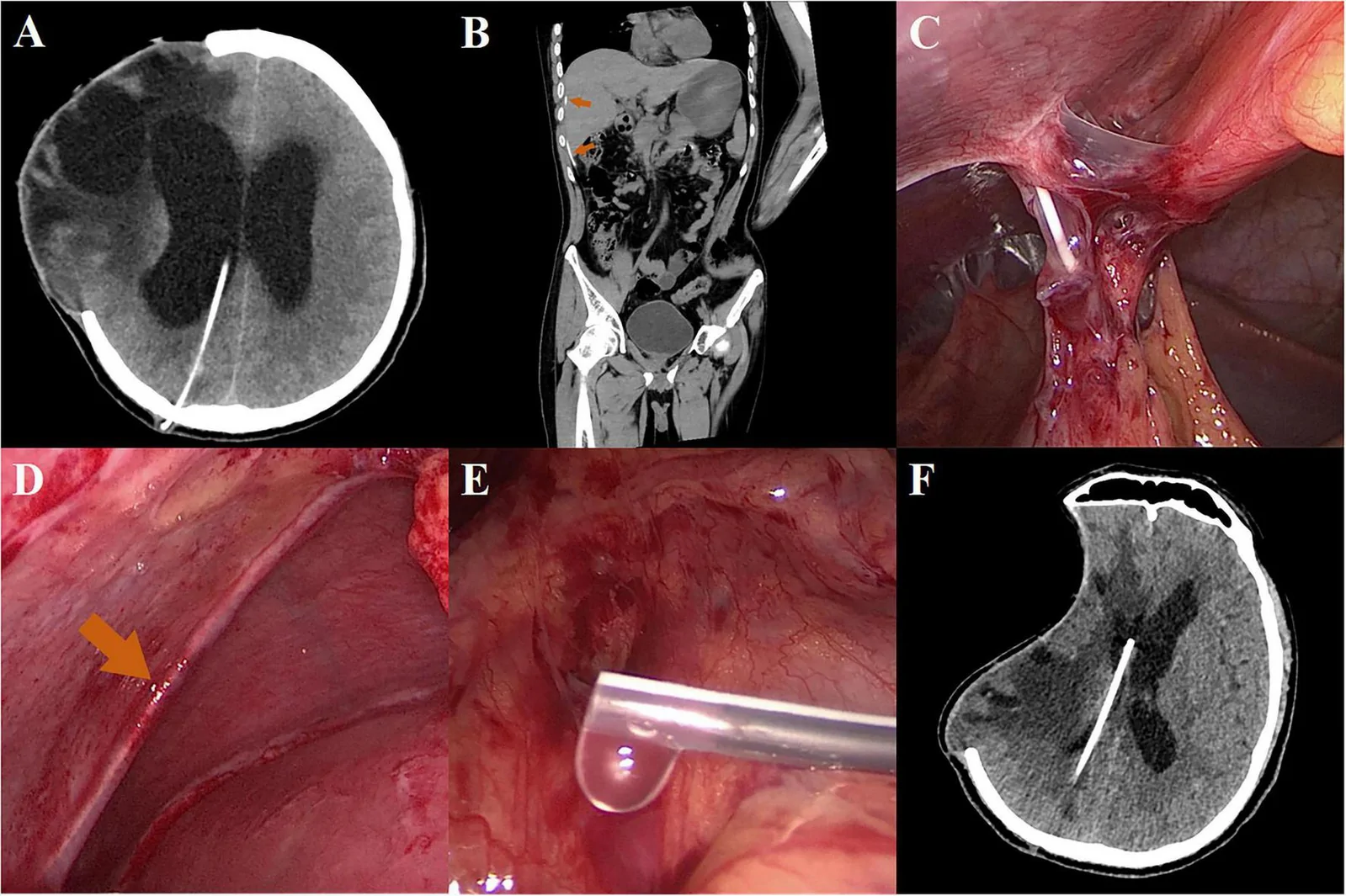
**(A)** Preoperative axial cranial CT. **(B)** Preoperative coronal abdominal CT showing the peritoneal catheter coursing along the right hepatic margin toward the inferior surface of the liver. **(C)** After adhesiolysis on the right side of the round ligament of the liver and exposure of the catheter tract, fluid egress was observed from the fibrous channel. **(D)** Dissection of the sheath along the hepatic surface. **(E)** The distal catheter was completely encased within a fibrous sheath and fixed to the parietal peritoneum. **(F)** Clear CSF outflow was observed after complete skeletonization of the shunt catheter.

A 30-years-old man underwent VP shunt placement for communicating hydrocephalus following intracerebral hemorrhage and subsequently developed recurrent shunt dysfunction (Supplementary Video 1). At the initial onset of his illness, the patient became comatose after alcohol consumption and was brought to the emergency department. Cranial computed tomography (CT) demonstrated a right basal ganglia–corona radiata–insular hemorrhage measuring approximately 75 mL with extension into the ventricular system and subarachnoid space. CT angiography revealed absence of bilateral posterior communicating arteries and vascular variation of the right anterior cerebral artery. Emergency hematoma evacuation, external ventricular drainage, and decompressive craniectomy were performed. Postoperative pathological examination suggested hemorrhage secondary to rupture of a vascular malformation. During recovery, the patient developed communicating hydrocephalus and subsequently underwent VP shunt insertion. The distal catheter was initially positioned in the left lower abdomen at the point symmetrical to McBurney’s point. Because the subsequent episodes of shunt dysfunction were attributed to suspected omental wrapping, distal catheter revisions were performed on days 11 and 28 after the initial shunt placement, respectively; however, each revision provided only transient improvement (Figure 1). Upon admission to our institution, physical examination demonstrated markedly increased tension over the decompressive cranial defect. Neuroimaging showed progressive ventricular enlargement. The patient remained afebrile during hospitalization. CSF analysis showed both a total cell count and a white blood cell count of 8 × 10^6^/L, a protein concentration of 371.7 mg/L, a glucose concentration of 3.6 mmol/L, and a lactate concentration of 2.4 mmol/L. No bacteria or fungi were identified on smear or culture. Preoperative cranial CT revealed severe ventriculomegaly accompanied by periventricular edema and focal external herniation of brain tissue through the cranial defect (Figure 2A). Abdominal CT demonstrated that the distal catheter entered the peritoneal cavity, reflected superiorly, and coursed along the right hepatic margin toward the inferior hepatic surface without evidence of surrounding fluid accumulation (Figure 2B).

**FIGURE 3:**
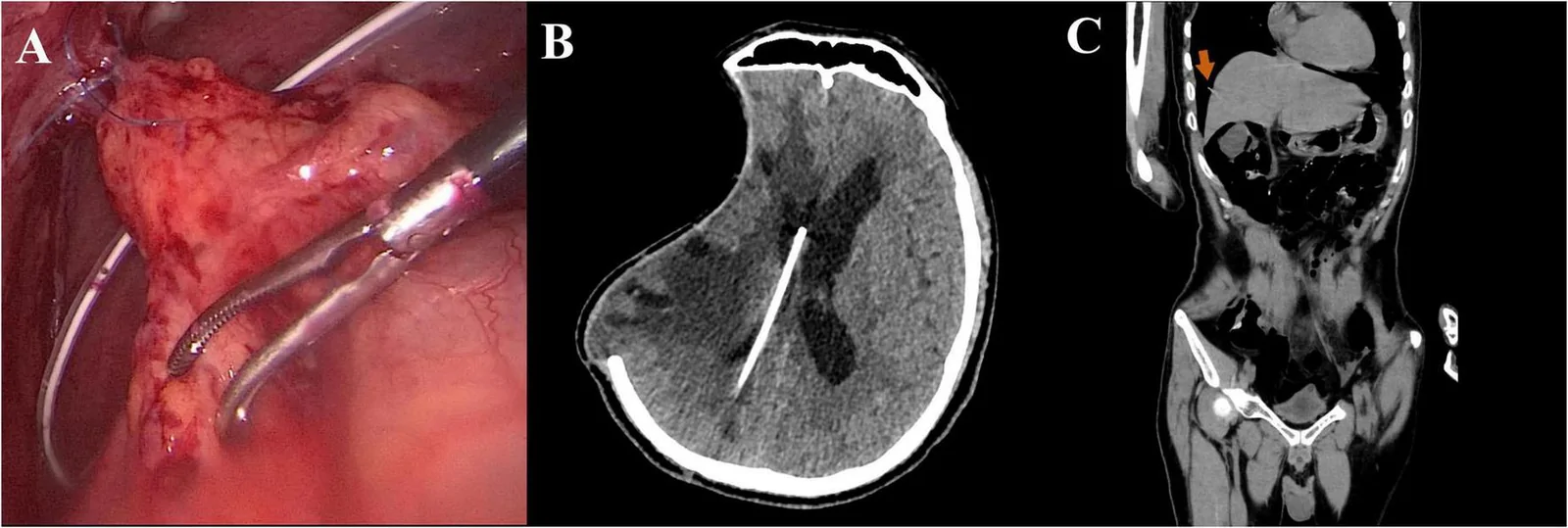
**(A)** Final position of the peritoneal catheter after revision. **(B)** Postoperative axial cranial CT. **(C)** Postoperative coronal abdominal CT.

Diagnostic laparoscopy was subsequently performed. Initial exploration revealed dense adhesions involving the greater omentum, transverse colon, and hepatic surface. The distal catheter entered the abdomen beneath the right costal margin, lateral to the round ligament of the liver, and demonstrated extensive adhesion to the transverse colon and omentum. The catheter then coursed along the diaphragmatic surface of the liver before extending inferiorly toward the hepatorenal recess. Because dense proximal adhesions limited direct visualization of the relationship between the omental component and the distal fibrotic lesion, intraoperative functional assessment was performed. Manual compression of the cranial shunt reservoir produced fluid egress from the adhesiolysed region adjacent to the round ligament of the liver (Figure 2C). This finding suggested continuity between the proximal omental adhesions and the distal fibrous sheath. Gentle traction allowed approximately 10 cm of catheter mobilization before spontaneous recoil occurred, suggesting dense distal fixation and possible negative pressure within the enclosed cavity. Further adhesiolysis over the hepatic surface exposed a complete fibrous sheath surrounding the distal catheter and firmly anchored to the parietal peritoneum (Figures 2D,E). After the fibrous sheath was incised at the site indicated by the yellow arrow in Figure 2D, the catheter was withdrawn and completely skeletonized. Subsequent compression of the cranial shunt reservoir produced clear CSF outflow from the distal catheter tip (Figure 2F). Inspection of the removed distal segment demonstrated a patent lumen without intrinsic obstruction. Further dissection confirmed that the fibrous sheath had formed a blind-ended dead space that effectively isolated both the catheter tip and side holes from the free absorptive surface of the peritoneal cavity. The fibrous sheath was completely detached laparoscopically from the parietal peritoneal surface. Two additional side holes were created in the distal catheter to optimize drainage. As part of our routine distal catheter revision technique, two additional side holes were created to reduce the risk of subsequent mechanical obstruction. The revised catheter was repositioned within the subphrenic space without being fixed to the peritoneal surface. Instead, the pre-existing fixation suture was used to restrict its range of motion within the subphrenic space, thereby avoiding persistent contact between the catheter and the parietal peritoneum (Figure 3A and Supplementary Video 1).

Postoperatively, the previously tense cranial defect became markedly sunken and soft.

Follow-up CT obtained on postoperative day 1 provided objective evidence of restored CSF drainage. Compared with preoperative imaging, the previously herniated brain tissue no longer protruded beyond the cranial defect. Ventricular enlargement was significantly reduced, accompanied by decreased periventricular edema (Figure 3B). The distal catheter remained in satisfactory position (Figure 3C). The patient experienced mild transient abdominal discomfort, which was presumed to be secondary to peritoneal irritation from CSF exposure and resolved after conservative treatment. At the 3-months follow-up, the patient remained clinically stable without recurrent shunt dysfunction or additional complications, and repeat cranial CT showed no recurrence of ventriculomegaly.

## 3 Discussion

This case demonstrates a unique mechanism of distal ventriculoperitoneal (VP) shunt failure and should not be simply described as omental wrapping (8). The obstructive lesion observed in this patient represented a hybrid structure: although the outer component involved the greater omentum, the more critical finding was the presence of an inner continuous dense fibrous sheath firmly fixed to and grossly continuous with the parietal peritoneum of the right upper abdominal wall. Review of the existing literature indicates that distal abdominal shunt failure is generally attributed to pseudocyst formation, adhesions, catheter migration, perforation, and omental wrapping (5–7). However, to our knowledge, this specific hybrid structure, characterized by continuity between proximal omental adhesions and a distal fibrous sheath fixed to the parietal peritoneum, has not previously been reported and may have important clinical implications. We speculate that the pathological process began with persistent contact between the catheter and the parietal peritoneal surface, resulting in focal mesothelial injury accompanied by reactive fibrosis. Within the relatively confined subphrenic space, the catheter may have become fixed in this location, allowing continuous exposure of the adjacent mesothelial layer to potentially irritating CSF. Together with the foreign-body reaction induced by the silicone catheter itself and chronic mechanical friction, these factors may have triggered local mesothelial-to-mesenchymal transition (MMT) and progressive fibrosis. Chronic mesothelial injury may activate profibrotic pathways, particularly transforming growth factor-β signaling, promoting the acquisition of a fibroblast-like phenotype and the deposition of collagen and other extracellular matrix components. These processes may ultimately have contributed to the formation of a dense encapsulating channel tightly fused with the abdominal wall (Figure 2D). Evidence from the peritoneal dialysis literature provides a pathophysiologically plausible framework for this hypothesis, in which MMT and progressive peritoneal fibrosis are recognized responses to chronic stimulation and biocompatibility-related exposure (9, 10). Previous case reports have also described localized fibrous encapsulation of peritoneal shunt catheters in the absence of overt infection. Histopathological findings in these reports included peritoneal tissue with a foreign-body reaction, hyalinized membranous tissue surrounded by fibrosis, fibrous tissue with minimal inflammatory-cell infiltration and a calretinin-positive mesothelial lining, and dense, predominantly acellular hyalinized fibrous tissue (11–13). These observations support the biological plausibility of a localized peritoneal fibrotic response to an indwelling catheter. However, the previously reported lesions were predominantly catheter-associated fibrous capsules and differed anatomically from the continuous hybrid omental–parietal peritoneal sheath fixed to the abdominal wall in the present case. The evidence from peritoneal dialysis and previous catheter-encapsulation reports should be regarded as biological analogies rather than direct proof of the cellular mechanism in this patient. In the present case, mechanistic confirmation was not possible because histopathological evidence was unavailable. The lesion was not anticipated preoperatively, and the intraoperative priority was to restore shunt function rather than obtain tissue for diagnostic analysis. Therefore, no specimen was preserved for histopathological examination. Consequently, the proposed MMT-related mechanism should be regarded only as a literature-supported and biologically plausible hypothesis that was not confirmed by direct histological evidence in this patient. The greater omentum was likely recruited secondary to the surgical incision and became adherent around the operative field, contributing an outer structural component to the encapsulation. Nevertheless, the principal barrier impairing CSF absorption remained the blind fibrotic cavity derived from the parietal peritoneum.

We acknowledge that this report has certain limitations, most notably the lack of histopathological confirmation. However, the following three independent and mutually supportive findings strengthen the credibility of our clinical interpretation: ① Laparoscopy directly demonstrated that the fibrous sheath was immobile and grossly continuous with the parietal peritoneum, a finding that differs substantially from the morphology of free omental wrapping. ② Intraoperative pump testing demonstrated no CSF outflow before incision of the sheath wall, suggesting that formation of a complete fibrous capsule resulted in total mechanical obstruction. ③ The rapid and marked postoperative radiological improvement, including resolution of cerebral herniation, reduction of ventricular size, and collapse of the cranial defect, confirmed that the identified obstruction was responsible for the shunt malfunction.

Conceptually, this lesion also differs from an abdominal pseudocyst. Pseudocysts represent reactive fluid collections surrounding the distal catheter (14, 15). In contrast, no obvious free fluid accumulation was identified preoperatively in this patient. Laparoscopic exploration demonstrated a solid, fixed, blind-ending fibrous sheath that physically isolated the catheter tip and side holes from the broader absorptive peritoneal surface. Simple omental wrapping usually involves relatively mobile omental tissue surrounding or obstructing the catheter without a continuous inner sheath fixed to the parietal peritoneum. In the present case, the fixed parietal attachment and the demonstrated continuity between the proximal omental adhesions and distal fibrous sheath distinguished the lesion from isolated omental wrapping. This distinction may account for the discrepancy between the relatively subtle preoperative imaging findings and the severe clinical manifestation of shunt malfunction. The patient remained afebrile during hospitalization. CSF analysis showed total and white blood cell counts of 8 × 10^6^/L, and bacterial and fungal smears and cultures were negative. These findings provided no evidence of an active shunt infection. Therefore, this lesion may be more consistent with a non-infectious peritoneal reactive process rather than classical pseudocyst formation. This distinction may explain why preoperative imaging findings were relatively inconspicuous despite severe clinical shunt dysfunction.

This case also provides several practical clinical implications. In patients with recurrent distal shunt dysfunction, particularly when abdominal imaging demonstrates persistent localization of the catheter tip within the right upper abdomen or subphrenic recess, surgeons should consider the possibility of focal abdominal wall encapsulation in addition to pseudocysts and omental wrapping.

**FIGURE 4:**
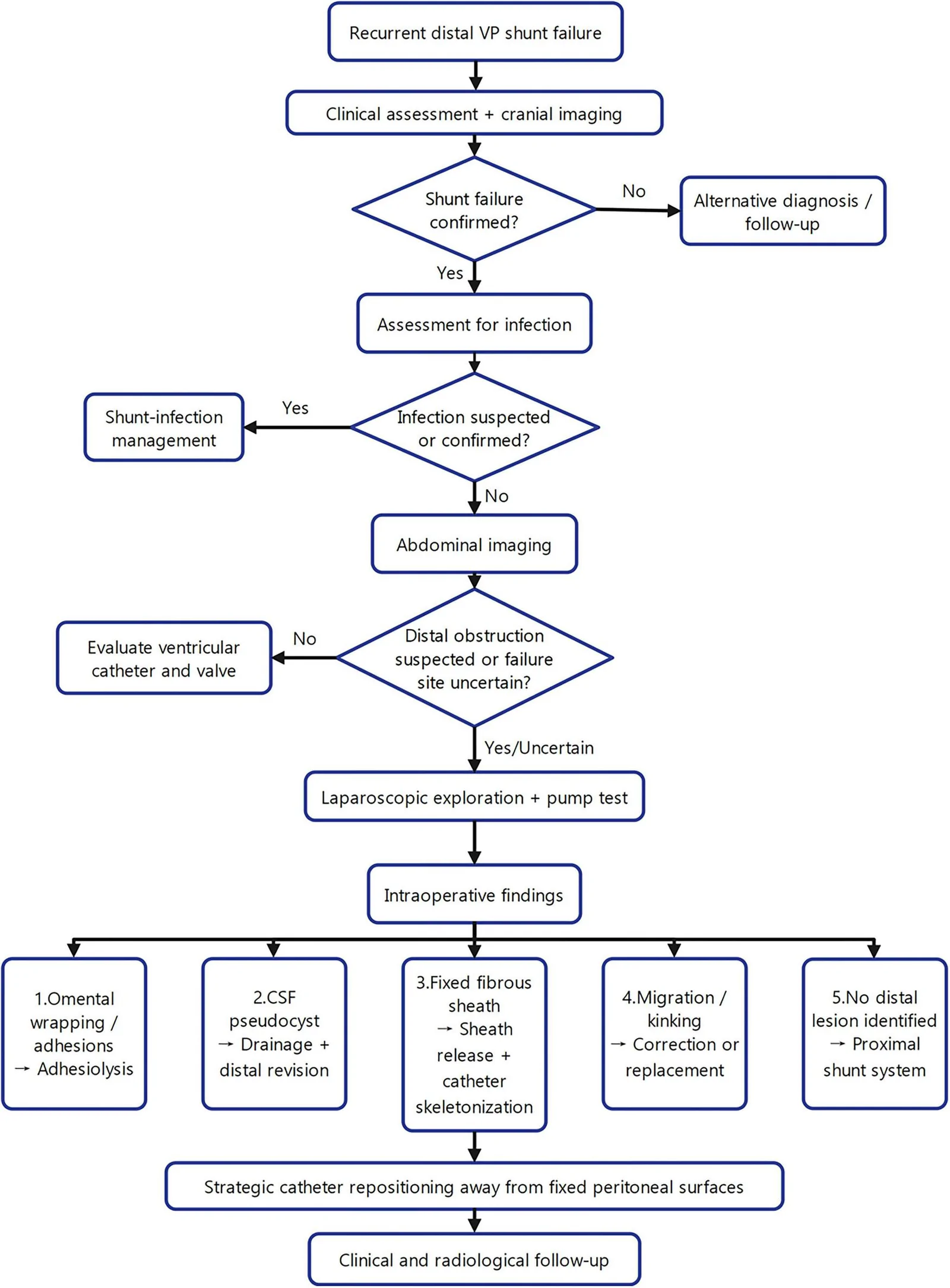
Stepwise approach to the management of recurrent distal ventriculoperitoneal shunt failure.

Laparoscopy offers unique advantages in the diagnosis and treatment of such lesions. It enables direct visualization of the catheter, confirmation of distal patency through pump testing, completion of adhesiolysis and fibrous sheath incision, and controlled repositioning of the catheter to locations with less persistent contact with the abdominal wall (16–18). Surgical management should target the underlying cause of obstruction. When it is difficult to determine whether malfunction originates from the distal abdominal component or the ventricular component, abdominal exploration should be prioritized to minimize patient morbidity. Based on the lessons from this case, we propose a stepwise approach to distal shunt dysfunction for the management of recurrent distal shunt failure (Figure 4).

Previous laparoscopic shunt studies have also supported placement of the distal catheter near the subphrenic space to preserve distal patency (19). Therefore, when an in-situ fibrous sheath caused by fixation to the parietal peritoneum is identified, the distal catheter should be intentionally repositioned within the subphrenic space while maintaining maximal separation from the fixed peritoneal surface. In the present case, the catheter remained within the subphrenic space, and the pre-existing fixation suture was used to restrict its range of movement without fixing the catheter directly to the peritoneal surface. When technically appropriate, the parietal peritoneum, greater omentum, or a hepatic ligament may serve as an anchor point for a loose limiting suture, provided that persistent contact between the catheter tip and a fixed peritoneal surface is avoided. As part of our routine distal catheter revision technique, two additional side holes were created to increase the available drainage surface and reduce the risk of subsequent mechanical obstruction. Because the size and spacing of these additional openings were not standardized, this technical modification should not be regarded as an evidence-based requirement. Although anti-adhesion barriers might theoretically reduce recurrent fixation or encapsulation, direct evidence supporting their prophylactic use around distal VP shunt catheters remains insufficient; therefore, their routine use cannot currently be recommended.

At the 3-months follow-up, the patient remained clinically stable without recurrent shunt dysfunction or additional complications, and repeat cranial CT showed no recurrent ventricular enlargement. However, the relatively short follow-up period remains a limitation, and longer observation is required to evaluate the durability of the catheter-repositioning strategy.

## 4 Conclusion

We report a rare but clinically significant cause of VP shunt malfunction characterized by the formation of a hybrid omental–parietal peritoneal fibrous sheath within the subphrenic space. The lesion created a sealed, blind-ending cavity that isolated the distal catheter from the absorptive peritoneal surface and resulted in recurrent distal shunt failure. Neurosurgeons managing unexplained or recurrent distal shunt dysfunction, particularly after repeated abdominal revisions or when conventional causes do not fully explain the malfunction, should consider this anatomical entity as a distinct mechanical cause of obstruction. In such cases, laparoscopic exploration may enable direct identification of the lesion, complete adhesiolysis and sheath release, and strategic repositioning of the catheter away from fixed peritoneal surfaces, thereby providing both diagnostic clarification and targeted treatment.

## Data Availability

The raw data supporting the conclusions of this article will be made available by the authors, without undue reservation.

## Supplementary material

Supplementary Video 1 Surgical video.

## References

[1] Isaacs AM Yang R Cadieux M Ben-Israel D Sader N Opoku-Darko M et al. Characteristics of shunt failure in 38,095 adult shunt insertion surgeries: a systematic review and meta-analysis. *Neurosurg Focus*. (2023) 54:E2. 10.3171/2023.1.FOCUS22637 37004137

[2] Hariharan P Sondheimer J Petroj A Gluski J Jea A Whitehead WE et al. A multicenter retrospective study of heterogeneous tissue aggregates obstructing ventricular catheters explanted from patients with hydrocephalus. *Fluids Barriers CNS*. (2021) 18:33. 10.1186/s12987-021-00262-3 34289858 PMC8293524

[3] Harischandra LS Sharma A Chatterjee S. Shunt migration in ventriculoperitoneal shunting: a comprehensive review of literature. *Neurol India*. (2019) 67:85–99. 10.4103/0028-3886.253968 30860103

[4] Anderson IA Saukila LF Robins JMW Akhunbay-Fudge CY Goodden JR Tyagi AK et al. Factors associated with 30-day ventriculoperitoneal shunt failure in pediatric and adult patients. *J Neurosurg*. (2019) 130:145–53. 10.3171/2017.8.JNS17399 29521592

[5] Simon TD Hall M Dean JM Kestle JR Riva-Cambrin J. Reinfection following initial cerebrospinal fluid shunt infection. *J Neurosurg Pediatr*. (2010) 6:277–85. 10.3171/2010.5.PEDS09457 20809713 PMC2953872

[6] Chopra I Gnanalingham K Pal D Peterson D. A knot in the catheter–an unusual cause of ventriculo-peritoneal shunt blockage. *Acta Neurochir* (2004) 146:1055–6. 10.1007/s00701-004-0320-6 15340821

[7] Ferreira Furtado LM Da Costa Val Filho JA Faleiro R Vieira JA Dos Santos AK. Abdominal complications related to ventriculoperitoneal shunt placement: a comprehensive review of literature. *Cureus.* (2021) 13:e13230. 10.7759/cureus.13230 33585146 PMC7877257

[8] Argo JL Yellumahanthi DK Ballem N Harrigan MR Fisher WS Wesley MM et al. Laparoscopic versus open approach for implantation of the peritoneal catheter during ventriculoperitoneal shunt placement. *Surg Endosc*. (2009) 23:1449–55. 10.1007/s00464-008-0245-x 19083058

[9] del Peso G Jiménez-Heffernan J Bajo M Aroeira L Aguilera A Fernández-Perpén A et al. Epithelial-to-mesenchymal transition of mesothelial cells is an early event during peritoneal dialysis and is associated with high peritoneal transport. *Kidney International*. (2008) 73:S26–33. 10.1038/sj.ki.5002598 18379544

[10] Aroeira LS Aguilera A Selgas R Ramírez-Huesca M Pérez-Lozano ML Cirugeda A et al. Mesenchymal conversion of mesothelial cells as a mechanism responsible for high solute transport rate in peritoneal dialysis: role of vascular endothelial growth factor. *Am J Kidney Dis*. (2005) 46:938–48. 10.1053/j.ajkd.2005.08.011 16253736

[11] Kano T. Fibrous capsule formation of the peritoneal catheter tip in ventriculoperitoneal shunt: two case reports. *Surg Neurol Int*. (2014) 5:S451–4. 10.4103/2152-7806.143275 25422787 PMC4235115

[12] Kano T Kawauchi H. Fibrous encapsulation of the peritoneal catheter in peritoneal shunt: case report. *Surg Neurol Int*. (2017) 8:132. 10.4103/sni.sni_420_16 28713635 PMC5502295

[13] Heaney J Johnson R. Ventriculoperitoneal shunt malfunction due to a fibrous capsule obstructing the peritoneal catheter. *Cureus*. (2020) 12:e6831. 10.7759/cureus.6831 32181074 PMC7051113

[14] Dabdoub CB Dabdoub CF Chavez M Villarroel J Ferrufino JL Coimbra A et al. Abdominal cerebrospinal fluid pseudocyst: a comparative analysis between children and adults. *Childs Nerv Syst*. (2014) 30:579–89. 10.1007/s00381-014-2370-2 24469949

[15] Mobley LW Doran SE Hellbusch LC. Abdominal pseudocyst: predisposing factors and treatment algorithm. *Pediatr Neurosurg*. (2005) 41:77–83. 10.1159/000085160 15942277

[16] Sheikhbahaei E Sabahi M Roy M Mandel M Adada BA Borghei-Razavi H. Letter to the editor regarding “laparoscopically assisted ventriculoperitoneal shunt placement is not cost-effective nor preventive for distal shunt malfunction”. *World Neurosurg*. (2022) 164:467–8. 10.1016/j.wneu.2022.04.114 35941776

[17] Mwachaka PM Obonyo NG Mutiso BK Ranketi S Mwang’ombe N. Ventriculoperitoneal shunt complications: a three-year retrospective study in a Kenyan national teaching and referral hospital. *Pediatr Neurosurg*. (2010) 46:1–5. 10.1159/000314050 20453556

[18] Patwardhan RV Nanda A. Implanted ventricular shunts in the United States: the billion-dollar-a-year cost of hydrocephalus treatment. *Neurosurgery* (2005) 56:139–44. 10.1227/01.neu.0000146206.40375.41 15617596

[19] Ding Q Wang J Fan H Jiang W Guo H Ji H et al. Introduction and comparision of three different fixation methods in the suprahepatic space in laparoscopy-assisted ventriculoperitoneal shunt for hydrocephalus. *Sci Rep* (2023) 13:6231. 10.1038/s41598-023-33566-5 37069252 PMC10110567

